# Plasma Exosomal-Derived SERPINA1 and GNAI2 Downregulation as Potential Diagnostic Biomarkers of Kawasaki Disease with Coronary Artery Aneurysms

**DOI:** 10.3390/ijms26062668

**Published:** 2025-03-16

**Authors:** Yang Zheng, Baoling Bai, Zhimiao Wei, Mingming Zhang, Qin Zhang, Xiaohui Li

**Affiliations:** 1 Department of Cardiovascular Medicine, Children’s Hospital Capital Institute of Pediatrics, Peking Union Medical College Graduate School, Beijing 100020, China; zhengyang201807@163.com; 2 Beijing Municipal Key Laboratory of Child Development and Nutriomics, Capital Institute of Pediatrics, Beijing 100020, China; baoxiang8802@126.com; 3 Department of Cardiovascular Medicine, Children’s Hospital Capital Institute of Pediatrics, Beijing 100020, China; weimiao77@163.com (Z.W.); zhangmm6526@163.com (M.Z.)

**Keywords:** Kawasaki disease (KD), coronary artery aneurysms (CAAs), exosome, proteomics

## Abstract

Kawasaki disease (KD) with coronary artery aneurysms (CAAs) is currently the primary cause of childhood acquired heart disease with an unclear pathogenesis. We established five groups for the discovery of differentially expressed proteins (DEPs): healthy control, febrile control, KD without CAAs, KD with small and medium CAAs, and KD with giant CAAs (*n* = 8 in each group). The validation of selected DEPs was conducted in another five groups (*n* = 4 in each group). We conducted comprehensive bioinformatics analyses to elucidate the functional roles of the DEPs in the groups of KD with CAAs and KD without CAAs. A total of 104 DEPs were identified in KD patients, which were primarily associated with complement-related pathways. A trend analysis of these 104 DEPs revealed 54 significantly changed DEPs associated with increased disease severity, which were primarily associated with G-protein-related functions. The alterations in α-1-antitrypsin short peptide (SERPINA1) and guanine nucleotide-binding protein G(i) subunit alpha-2 (GNAI2), which were selected from complement-related and G-protein-related pathways, respectively, were validated by Western blotting, and they were significantly decreased in KD patients with vs. without CAAs. In addition, we conducted an analysis of the DEPs in the groups of KD with CAAs and KD without CAAs, separately. There were 91 DEPs specifically expressed in KD patients with CAAs, associated with the neutrophil extracellular trap and complement pathways, while 16 DEPs were specific to those without CAAs, associated with viral infection and immunity pathways. Additionally, for DEPs among different severities of CAAs, there were 102 DEPs in KD patients with small and medium CAAs, associated with complement pathways and platelet activation pathways, whereas 34 DEPs were specific to giant CAAs, associated with the Rap1 signaling pathway and cell functions. In conclusion, this study provides plasmatic exosomal protein profiles in KD patients with CAAs, suggesting that SERPINA1 and GNIA2 might serve as novel potential diagnostic biomarkers for KD with CAAs.

## 1. Introduction

Kawasaki disease (KD), also known as mucocutaneous lymph node syndrome, is an acute immune-mediated vasculitis of unknown etiology that predominantly affects children under the age of 5 years old [1]. Coronary artery lesions (CALs) resulting from KD are the leading cause of acquired heart disease among children in developed countries [2,3]. CALs include coronary artery dilatations (CADs) and coronary aneurysms (CAAs). CAAs may lead to complications such as thrombosis, stenosis, and myocardial infarction, which severely impact the survival and development of young children [1,4]. The incidence of CALs was 20–25% in untreated KD patients. Despite being treated with intravenous immunoglobulin (IVIG), 3–5% KD patients still suffer from CAAs [5,6], and the mechanisms underlying KD with CAAs remain unclear.

Proteomics is dedicated to investigating the expression, function, and interactions of all proteins in cells, tissues, or the body under specific physiological or pathological conditions. In recent years, proteomics and multi-omics analyses have significantly advanced the understanding of cardiovascular diseases [7,8,9,10,11,12,13]. A study focusing on proteomics in KD has demonstrated that serum proteomic profiling identified 29 differentially expressed proteins (DEPs) in KD patients before and after IVIG therapy [14]. Plasma proteomic profiling has identified SERPINE1 as a potential biomarker associated with CALs in KD [15]. Serum proteomic profiling has also revealed 1879 DEPs during the acute and recovery phases of KD [16]. Proteomic analyses of urine and leukocytes have been conducted to discover novel biomarkers for KD [17,18,19]. These studies underscore the essential role of proteomics in mapping the protein expression profiles of KD patients and identifying diagnostic biomarkers. However, these proteomic studies were predominantly concentrated on the DEPs in bodily fluids. The role of proteins in specific components of blood, particularly those within plasmatic extracellular vesicles, in KD patients with and without CAAs has rarely been reported.

Exosomes are nanoscale extracellular vesicles with a lipid bilayer, typically measuring 50 to 100 nm in diameter. They play a crucial role in intercellular signaling and physiological functions through their stable cargos such as microRNAs, DNAs, lipids, and proteins [20,21]. Exosomes are found in various extracellular fluids throughout the body, such as blood, saliva, urine, cerebrospinal fluid, breast milk, and amniotic fluid [20]. Previous studies have demonstrated that serum exosomal microRNAs could serve as candidate diagnostic biomarkers for KD through RNA sequencing [22,23,24], suggesting the significant role that exosome cargos might play in KD. The roles of exosomes and proteomes in cancer, inflammation, and cardiovascular diseases have been reported [25,26,27]. As for the serum exosomal proteins of KD patients, Zhang et al. revealed 69 and 59 DEPs in KD patients before and after IVIG therapy, respectively, compared with the control group via two-dimensional electrophoresis (2-DE), and the complement system and innate immune response were enhanced in KD. This study might help in evaluating the therapeutic effects of IVIG therapy in KD patients and enhancing the understanding of IVIG therapy [28]. Another study reported 38 DEPs between KD patients with CADs and healthy children, which were associated with the acute inflammatory response, defense response, complement activation, and humoral immune response, providing a map of serum exosome proteins in the pathogenesis of CADs [29]. In addition, Xie et al. identified 32 DEPs in CAA patients, compared with healthy children, and they were associated with the host immune response, inflammation, apoptotic process, developmental process, and biological adhesion process, which provided a diagnostic clue for CAAs [30]. These findings suggest that exosome proteins may be involved in the CAA pathogenesis, but the difference between KD patients with and without CAA was not reported. Moreover, few studies focusing on KD with different severities of CAAs, the most serious complication and the most important factor affecting KD prognosis, have been reported.

In the present study, we investigated potential plasmatic exosomal proteomic profiles in the KD groups with and without CAAs, as well as in KD groups with different severities of CAAs. This is expected to provide a clue for the early potential diagnostic biomarkers for KD with CAAs and a basis for further discovery.

## 2. Results

### 2.1. Clinical Characteristics of Children with KD

The baseline characteristics of the KD patients and the controls are listed in Table 1. In the 40 subjects in the discovery group, fifty percent were boys in each subgroup. There was no significant difference among groups (*p* > 0.999). The average ages in the KD-1, KD-2, and KD-3 groups are listed in Table 1. There was no significant difference in age among groups (*p* = 0.954). Z-scores in the KD-2 and KD-3 groups were 5.87 ± 2.87 and 13.23 ± 2.63, respectively, distinguishing the different severity of CAAs (*p* < 0.001). The inflammatory factors of KD patients without and with CAAs are listed in Table 1 and Table 2. The numbers of white blood cells (WBCs) in the KD groups with and without CAAs were 11.86 ± 5.99 and 13.01 ± 4.83 ×10^9^/L, respectively. There was no significant difference among groups (*p* = 0.641). The numbers of neutrophils (NEUT) in the KD groups with and without CAAs were 6.90 ± 4.75 and 8.15 ± 3.02 ×10^9^/L, respectively. There was no significant difference among groups (*p* = 0.506). Notably, the numbers of platelets (PLT) in the KD groups with and without CAAs were 556.00 ± 210.05 and 339.63 ± 102.75 ×10^9^/L, respectively. There was a significant difference among groups (*p* = 0.026), suggesting PLT activation in KD with CAAs. Furthermore, the numbers of PLT in the KD-1, KD-2, and KD-3 groups were 339.63 ± 102.75, 487.00 ± 210.05, and 625.00 ± 267.02 ×10^9^/L, respectively, highlighting the role of PLT activation in CAAs. The C-reactive protein (CRP) levels in the KD groups with and without CAAs were 24.50 (2.83,65.89) and 59.60 ± 32.28 mg/L, respectively. There was no significant difference among groups (*p* = 0.136). All patients did not have a family history of KD or other diseases.

In the 20 subjects of the validation group, seventy-five percent were boys. There was no significant difference among groups (*p* > 0.999). The average ages in the KD-1, KD-2, and KD-3 groups are listed, with no significant difference among groups (*p* = 0.972). Z-scores in the KD-2 and KD-3 groups were 6.23 ± 2.85 and 11.96 ± 2.09, respectively, distinguishing the different severities of CAAs (*p* = 0.018). The inflammatory factors of KD patients are listed in Table 1 and Table 2. The numbers of WBC in the KD groups with and without CAAs were 11.98 ± 3.70 and 14.97 ± 2.66 ×10^9^/L, respectively. There was no significant difference among groups (*p* = 0.185). The numbers of NEUT in the KD groups with and without CAAs were 7.30 ± 3.36 and 9.05 ± 3.15 ×10^9^/L, respectively. There was no significant difference among groups (*p* = 0.408). The numbers of PLT in the KD groups with and without CAAs were 470.75 ± 184.12 and 513.00 ± 62.80 ×10^9^/L, respectively. There was no significant difference among groups (*p* = 0.683). The CRP levels in the KD groups with and without CAAs were 73.18 ± 65.75 and 34.64 ± 23.56 mg/L, respectively. There was no significant difference among groups (*p* = 0.291). All patients did not have a family history of KD or other diseases.

### 2.2. Isolation and Validation of Plasma Exosomes

**Figure 1:**
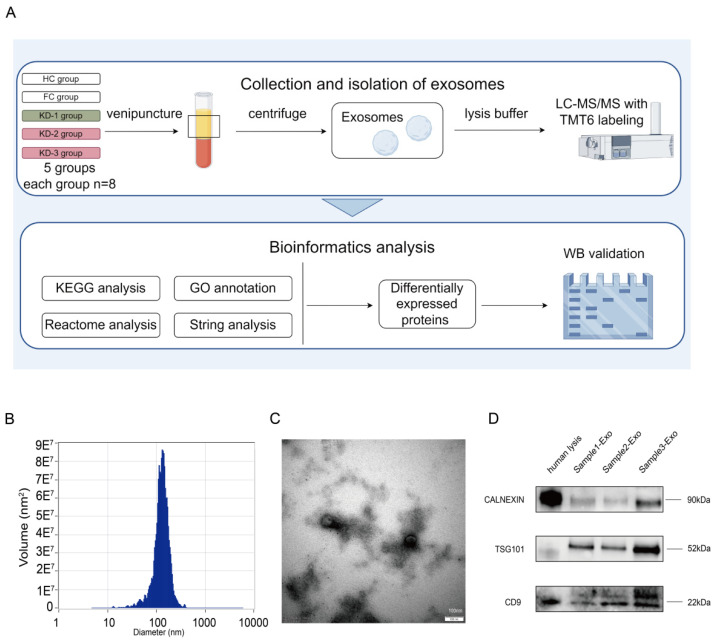
Flowchart and confirmation of plasma exosomes. (**A**) Flow diagrams of study design, classification of recruited children, and bioinformatics analysis workflow (image created with Figdraw 2.0, https://www.figdraw.com/static/index.html#/ and published with permission). KD-1: KD patients without CAAs, KD-2: KD patients with small and medium CAAs, KD-3: KD patients with giant CAAs. KD patients without and with CAAs are highlighted in green and pink, respectively. (**B**) Characterization by NTA of exosomes isolated from plasma of subjects, showing centralized range of diameters. (**C**) Transmission electron microscopy image of exosomes isolated from plasma of subjects. (**D**) Western blot of exosome protein markers (exosome markers CD9 and TSG101 and cell organelle marker CALNEXIN) in healthy children plasma and isolated exosomes. Equal amounts of protein were loaded in each lane.

The study design is illustrated in flow diagrams in Figure 1A. Initially, we analyzed the exosomes isolated from plasma samples. Nanoparticle Tracking Analysis (NTA) indicated that the diameter of the spherical vesicles ranged from approximately 40 to 150 nm among our subjects (Figure 1B), consistent with the previously reported size range of exosomes. Transmission electron microscopy further confirmed the presence of circular vesicles and peripheral membranous structures (Figure 1C). To validate our exosome isolation, we performed WB analysis using three protein markers: the exosome-specific markers CD9 and TSG101, and the cellular organelle marker CALNEXIN. An equal amount of protein was loaded into each lane. The results demonstrated that the exosome markers were significantly enriched in the isolated exosomes compared to the human cell lysate, whereas CALNEXIN, serving as a negative control, exhibited reduced enrichment in the isolated exosomes (Figure 1D). These findings confirmed the successful purification of intact exosomes from human plasma.

### 2.3. Expression Patterns of the Exosomal Proteome in KD Patients Compared with the HC Group

**Figure 2:**
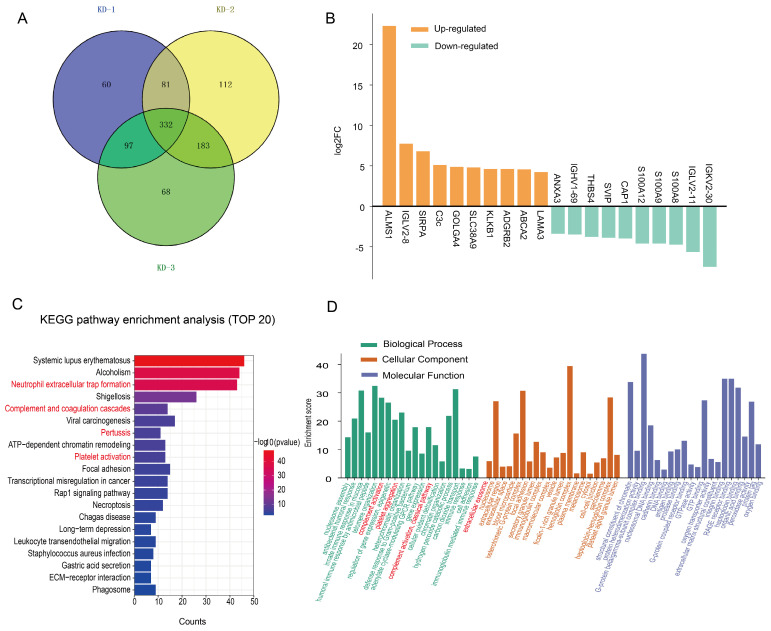
Protein expression profiles of plasma exosomes in KD patients compared with the HC group. (**A**) Venn diagram showing the number of DEPs in KD patients; 332 overlapped exosomal DEPs were commonly expressed among three subgroups. (**B**) The top 10 up-/downregulated proteins in the KD patients. (**C**) KEGG pathway enrichment analysis of 332 exosomal DEPs expressed in KD patients, showing the top 20 enriched pathways. (**D**) GO annotations for 332 exosomal DEPs expressed in KD patients. The red font referred to the pathways we focused and mentioned.

In order to reveal the expression characteristics of DEPs in KD patients, we performed an overall analysis of KD patients with and without CAAs. A total of 995 proteins were identified across all groups. We compared the expression of exosomal proteins in KD patients with those in the HC group. The KD-1, KD-2, and KD-3 groups exhibited 570, 708, and 680 DEPs, separately (Figure S1A). A Venn diagram analysis of these three groups revealed that 332 DEPs were commonly expressed among the KD-1, KD-2, and KD-3 groups, indicating their potential association with the pathophysiology of KD (Figure 2A). The top 10 DEPs in KD patients were ALMS1, C3c, S100A9, S100A8, etc. (Figure 2B). Upregulated proteins were related to complement–coagulation cascades, and downregulated proteins were linked to interleukin signaling and neutrophil degranulation.

A functional bioinformatics analysis of these 332 proteins was conducted, highlighting the primary pathways of DEPs in KD patients compared with the HC group. KEGG pathway enrichment analysis demonstrated that these DEPs were primarily enriched in pathways related to complement and coagulation cascades, as well as platelet activation, suggesting a potential link to KD (Figure 2C). Reactome and Wiki pathway enrichment analysis provided further insights. Apart from complement–coagulation cascade pathways, focal adhesion and gene modification pathways were enriched (Figure S1B,C). GO annotation of these 332 DEPs further revealed that they were mainly enriched extracellular exosomes and in pathways related to complement activation, platelet aggregation, and immune response (Figure 2D). These functions hold clinical relevance in the pathogenesis of KD.

The analysis revealed an overall profile of bioinformatics and pathway enrichment in KD patients. On this basis, we further explored the bioinformatic functions of specific DEPs expressed in KD patients.

### 2.4. Expression Patterns of the Exosomal Proteome in KD Patients Compared with the FC Group

In the early phase of KD, patients typically present with fever. To identify exosomal DEPs that can differentiate KD patients from those with febrile conditions, we recruited the FC group for comparison. Following the analysis of the 332 DEPs in KD patients, we investigated the comparative expression profiles between KD patients and the FC group.

**Figure 3:**
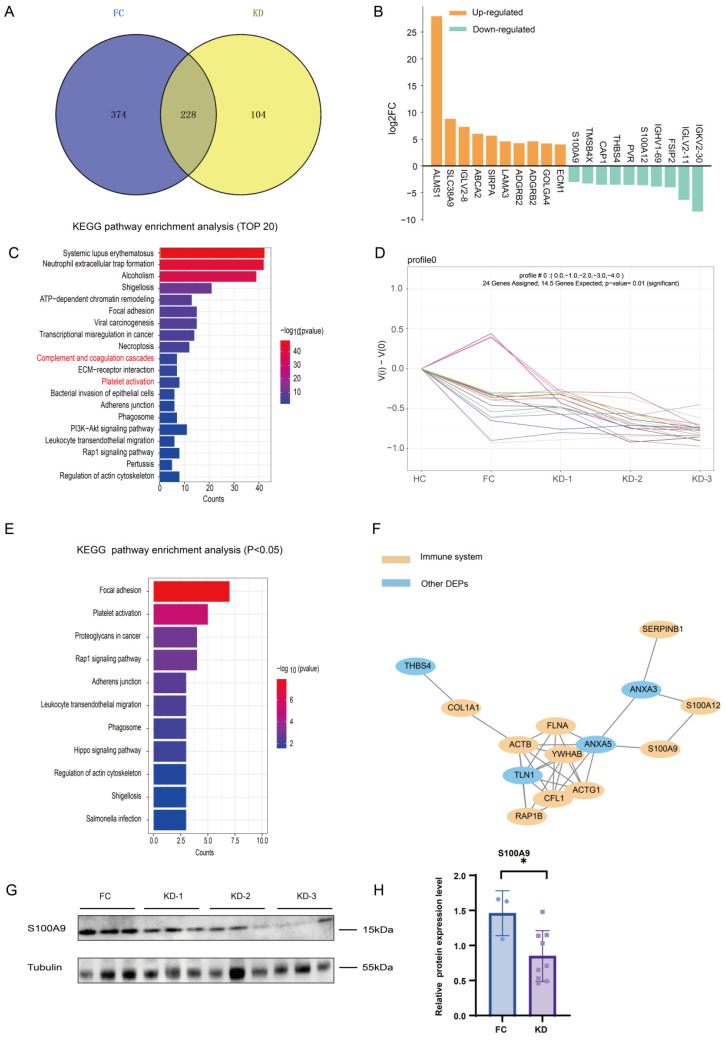
Protein expression profiles of plasma exosomes expressed in KD patients compared with the FC group. (**A**) Venn diagram showing the number of DEPs in the KD group compared with the FC group. (**B**) The top 10 up-/downregulated proteins both in KD patients and in the FC group. (**C**) KEGG pathway enrichment analysis of 228 expressed exosomal DEPs in the KD group, showing the top 20 enriched pathways. (**D**) Line graph showing expression changes in 24 exosomal DEPs with downregulated expression. (**E**) KEGG pathway enrichment analysis of 24 downregulated exosomal DEPs in Profile 0, showing all the enriched pathways with significant differences (*p* < 0.05). (**F**) STRING analysis of the PPI network among the 24 exosomal DEPs. (**G**) WB analysis to validate the expression level of S100A9 in KD patients and the FC group, revealing significant differences in KD patients compared to the HC group. The expression levels were normalized to α-Tubulin. (**H**) A bar chart showing the expression level of S100A9 in KD patients and the FC group. * indicates *p* < 0.05. The red font referred to the pathways we focused and mentioned.

The FC group exhibited 602 DEPs compared with the HC group (Figure S2A). A Venn diagram analysis revealed that there were 228 DEPs both in KD patients and the FC group and 104 DEPs expressed in KD patients (Figure 3A). The top 10 DEPs in the 228 DEPs in KD patients and the FC group were ALMS1, S100A9, S100A12, etc. (Figure 3B). Upregulated proteins were related to cell–cell communication, and downregulated proteins were linked to platelet degranulation and the immune system. The KEGG pathway enrichment analysis of these 228 DEPs revealed that pathways related to complement and coagulation cascades, and platelet activation and the Rap1 signaling pathway were enriched, aligning with the analysis of the 332 proteins presented in Figure 3C. The results of Reactome, Wiki pathway enrichment analysis, and GO annotation were similar to those in the 332 DEPs (Figure S2B–D), indicating that these pathways were significantly enriched in febrile and immune diseases. We further conducted a trend analysis based on the expression levels. The analysis revealed that, among the 228 DEPs, 24 proteins exhibited significantly changed expressions, which were associated with increased disease severity (Figure 3D). The KEGG pathway enrichment analysis of these 24 DEPs revealed that they were related to infection and cell function pathways (Figure 3E). The results of Reactome, Wiki pathway enrichment analysis, and GO annotation of these 24 DEPs were shown in Figure S2E–G, indicating that these pathways were significantly enriched in immune response. STRING analysis demonstrated protein interactions among 24 DEPs, as illustrated in Figure 3F, and most DEPs in the PPI network were linked with the immune system, including S100 family proteins.

Based on the complement-related pathways we enriched, we selected S100A9, one of the DEPs in the 24 changed proteins, as well as one of the top 10 DEPs in KD patients, for validation of its expression via WB analysis (Figure 3G, H). The expression trend of S100A9 was consistent with the LC-MS/MS result. The WB analysis revealed that the expression of S100A9 was significantly lower in KD patients compared with the FC group (*p* < 0.05), suggesting the role of S100A9 in KD patients compared with the FC group.

These results revealed the proteomic profile in KD patients compared with the FC group. In this section, we discovered and validated the DEPs expressed both in KD patients and the FC group. Next, we focused on the 104 specific DEPs in KD patients, including KD patients without and with CAAs, but not expressed in the FC group.

### 2.5. Expression Patterns of the Exosomal Proteome Expressed in the KD Groups with and Without CAAs

Following the results above, there were 104 DEPs specifically expressed in KD patients compared with the FC group (Figure 3A). We then analyzed these DEPs in KD patients, including the KD groups with and without CAAs in our study. The expression trend of these 104 proteins was further analyzed to investigate whether DEPs with different expression levels could distinguish KD complicated with CAAs. In addition to the analysis of 104 DEPs, we also conducted a categorization to analyze specific DEPs in the KD-1 (KD patients without CAAs) and KD-2 + KD-3 (KD patients with CAAs) groups to elucidate the expression profile and biological function in the KD patients with and without CAAs, respectively, in this section.

**Figure 4:**
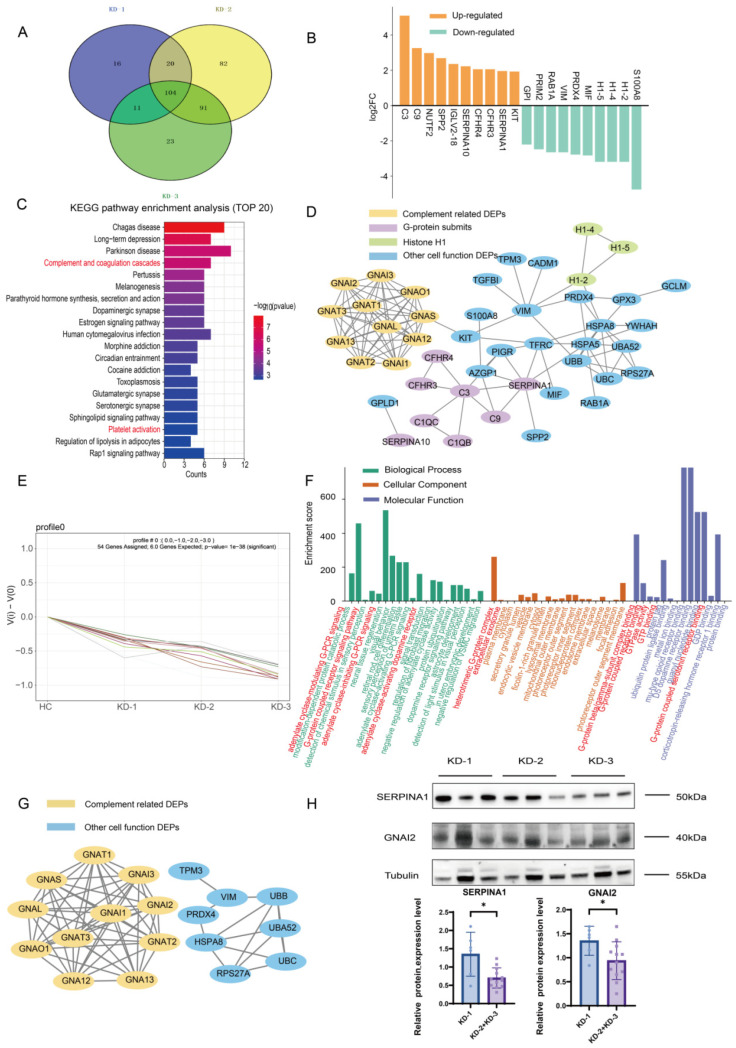
Protein expression profiles of plasma exosomes expressed in the KD groups without and with CAAs. (**A**) A Venn diagram showing the number of DEPs in the KD-1, KD-2, KD-3, and FC groups. (**B**) The top 10 up-/downregulated proteins in KD patients without and with CAAs. (**C**) KEGG pathway enrichment analysis of 104 expressed exosomal DEPs in KD patients, showing the top 20 enriched pathways. (**D**) STRING analysis of the PPI network among the 104 exosomal DEPs. (**E**) A line graph showing the expression changes in 54 exosomal DEPs with downregulated expression in Profile 0. (**F**) GO annotations for 54 exosomal DEPs in Profile 0. (**G**) STRING analysis of the PPT network for the 54 exosomal DEPs. (**H**) WB analysis of SERPINA1 and GNAI2 in the KD groups without and with CAAs; 2 DEPs revealed significant differences. The expression levels were normalized to α-Tubulin. The bar chart shows the expression level. * indicates *p* < 0.05. KD-1 group: KD patients without CAAs; KD-2 + KD-3 groups: KD patients with CAAs. The red font referred to the pathways we focused and mentioned.

A Venn diagram showed the number of DEPs and protein overlap between the KD-1, KD-2, and KD-3 groups in detail (Figure 4A). The top 10 DEPs among the 104 DEPs in KD patients were C3, S100A8, and α-1-antitrypsin short peptide (SERPINA1), etc. (Figure 4B). Upregulated proteins were related to complement and coagulation cascades and the immune system, and downregulated proteins were linked to cell death. A functional bioinformatics analysis of these 104 proteins was conducted, highlighting the primary pathways of DEPs expressed in the KD groups compared with the FC group. KEGG and Reactome pathway enrichment analysis revealed that these proteins were mainly enriched in pathways related to complement–coagulation cascades, which was our primary focus (Figure 4C and Figure S3A). Platelet activation, the pathway related to platelet function, was also enriched in the KEGG analysis of KD patients, suggesting the role of platelets in KD pathology, which was associated with the laboratory data in that the counts of platelets were higher in KD patients with CAAs. In the Wiki pathway enrichment analysis, complement system pathways were abundantly enriched, as well as G-protein signaling and other metabolic-related pathways (Figure S3B). GO annotation revealed that these DEPs were primarily enriched in functions related to complement activation, as well as G-protein-coupled receptor signaling and GTP-associated functions, highlighting the involvement of the G-protein in KD (Figure S3C). STRING analysis demonstrated protein interactions among the 104 DEPs, especially the protein interactions of complement proteins, as well as G-proteins and their submit family, as illustrated in Figure 4D. These findings suggested the importance of complement-related pathways in KD patients. Among the DEPs, SERPINA1 was highlighted as it belonged to complement pathways in KD patients compared with the FC group, which might be directly linked to alterations in immune function and the underlying mechanisms of KD due to its role in the inflammatory response, as reported.

To identify proteins that potentially exhibited differential roles in KD patients based on their expression levels, we next conducted a trend analysis, which revealed that among the 104 DEPs, 54 exhibited a significantly changed expression, associated with increased disease severity (*p* < 0.05), indicating that the changed protein expression levels were associated with increased disease severity (Figure 4E). We performed a functional analysis of these 54 proteins. As depicted in Figure S3D,E, KEGG and Reactome pathway analysis revealed an enrichment of platelet activation, disease regulation, gene modification, and signal transduction pathways. The GO annotation highlighted the enrichment of functions associated with G-proteins and GTPases, suggesting their involvement in G-protein signaling pathways. These functions were expressed in KD patients and demonstrated potential for differentiating the severity of the disease (Figure 4F). The results of Wiki pathway analysis are presented in Figure S3F, with G-protein signaling and signal transduction pathways shown to be enriched. STRING analysis demonstrated protein interactions among the 54 DEPs. The G-protein-related DEPs were closely linked, as illustrated in Figure 4G. Since guanine nucleotide-binding protein G(i) subunit alpha-2 (GNAI2) existed in all G-protein-related functions and the top 20 KEGG pathways, we then verified the GNAI2 expression level in the KD groups with and without CAAs.

Consequently, we proceeded to validate the data by WB analysis for the two DEPs: SERPINA1 and GNAI2 (Figure 4H). The expression trend of SERPINA1 was consistent with the LC-MS/MS result. The expression of SERPINA1 was lower in the KD-2 + KD-3 groups and showed a significant difference from the KD-1 group (*p* < 0.05), suggesting a potential role of SERPINA1 in KD with CAA patients. The expression trend of GNAI2 was similar. It was consistent with the LC-MS/MS result, its expression was lower in the KD-2 + KD-3 groups, and it showed a significant difference from the KD-1 group (*p* < 0.05). The expression pattern suggested that GNAI2 might be involved in the pathogenesis of KD and CAA patients. The WB analysis showed that the expression levels of these two DEPs were significantly lower in KD patients with vs. without CAAs, which might assist in the identification of CAAs.

In addition to analyzing DEPs with varying expression trends in KD patients, we also conducted separate analyses of DEPs specifically expressed in the presence or absence of CAAs. This approach allowed us to develop a comprehensive profile of the exosomal protein expression associated with CAAs.

**Figure 5:**
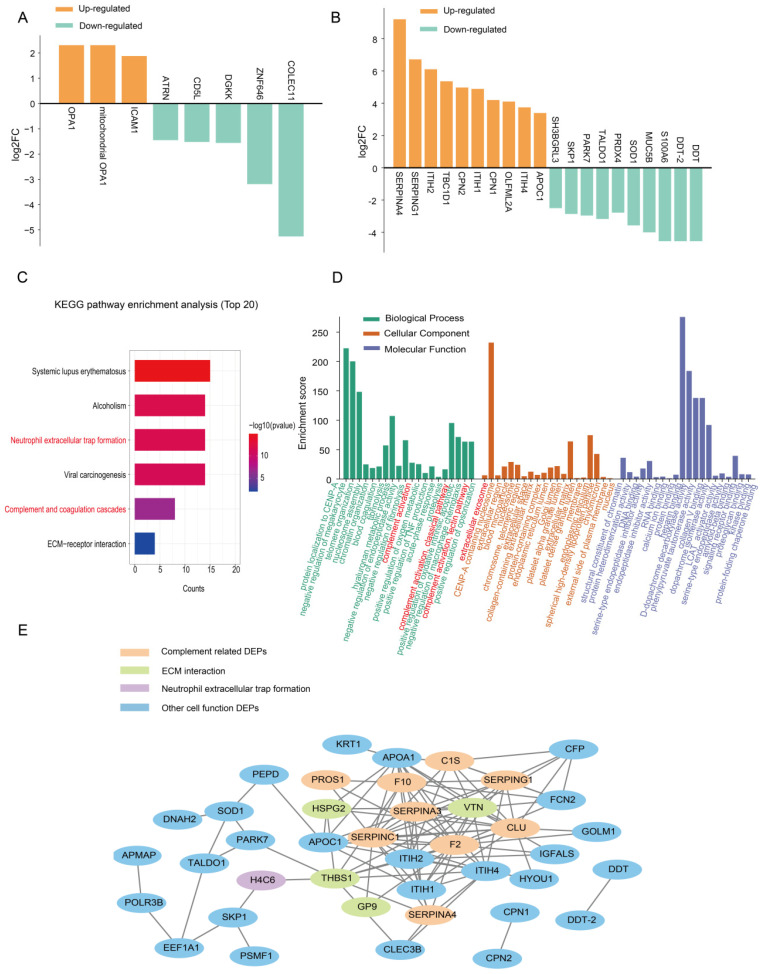
Protein expression profiles of plasma exosomes in the KD groups without and with CAAs separately. (**A**) The top up-/downregulated proteins in KD patients without CAAs, showing all upregulated DEPs and the top five downregulated DEPs. (**B**) The top 10 up-/downregulated proteins in KD patients with CAAs. (**C**) KEGG pathway enrichment analysis of 91 exosomal DEPs expressed in the KD-2 + KD-3 groups, showing all enriched pathways. (**D**) GO annotations for 91 exosomal DEPs expressed in the KD-2 + KD-3 groups. (**E**) STRING analysis of the PPT network among the 91 exosomal DEPs. The red font referred to the pathways we focused and mentioned.

We compiled the DEPs from the KD-2 and KD-3 groups, examining both their union and intersection. This analysis enabled us to identify the DEPs expressed in the KD-1 group, as well as those in the KD-2 and KD-3 groups. The Venn diagram revealed that 91 DEPs were specifically expressed in the KD-2 + KD-3 groups, and 16 DEPs were specifically expressed in the KD-1 group (Figure 4A), suggesting the different proteins affected in the presence or absence of CAAs. The functional bioinformatics analyses showed the pathways of DEPs in the KD groups with and without CAAs, respectively. The top DEPs (Figure 5A,B) and pathway analysis, as well as the PPI network, were discussed. For the 91 DEPs in KD patients with CAAs, the top 10 DEPs were SERPINA4, SERPING1, S100A6, etc. (Figure 5B). Upregulated proteins were related to complement cascade and platelet degranulation. Downregulated proteins were linked to interleukin signaling and the immune system. KEGG pathway enrichment analysis revealed that these proteins were enriched in neutrophil extracellular trap information and complement and coagulation cascades, indicating the significance of pathways in the pathogenesis of CAAs (Figure 5C). The results of the Reactome and Wiki pathway enrichment analysis are presented in Figure S4A,B, respectively, showing complement and coagulation cascades and histone modifications pathways. GO annotation further indicated that these proteins are involved in functions related to protein localization and organelle activity, and the complement activation and classical pathways in the extracellular exosome were enriched (Figure 5D). STRING analysis demonstrated protein interactions among 91 DEPs (Figure 5E), showing the PPI network of DEPs in complement pathways and extracellular matrix functions.

For the 16 DEPs in KD patients without CAAs, the top DEPs were OPA1, ICAM1, COLEC11, etc. (Figure 5A). Upregulated proteins were related to leukocyte adhesion, and downregulated proteins were linked to innate immunity. KEGG and Reactome pathway enrichment analysis revealed that these DEPs were primarily enriched in pathways related to viral infection and immunity (Figure S4C,D). Additionally, GO annotation highlighted the cell adhesion enriched by these proteins, as shown in Figure S4E.

These results revealed the proteomic profiles of KD patients with CAAs and KD patients without CAAs, suggesting the potential pathological mechanisms of CAAs. The two DEPs, SERPINA1 and GNAI2, might have the potential to serve as potential diagnostic biomarkers for KD with CAAs.

### 2.6. Expression Patterns of the Exosomal Proteome Expressed in KD Patients with Different Severities of CAAs

To further discover the pathological mechanisms of different severities of CAAs, we continued to elucidate the expression profile and biological function of specific exosomal proteins in KD patients with different severities of CAAs, and we conducted an additional categorization to analyze DEPs in the KD-2 and KD-3 groups, respectively. The Venn diagram revealed 102 and 34 DEPs that were specifically expressed in the KD-2 and KD-3 groups (Figure S5A), respectively, suggesting that different proteins are affected depending on the severity of CAAs.

For the 102 DEPs in KD patients with small and medium CAAs, the top 10 DEPs in the KD-2 group were SERPIND1, C5, etc. (Figure S5B). Upregulated proteins were related to complement cascades, and downregulated proteins were linked to platelet degranulation and the immune system. KEGG and Reactome pathway enrichment analysis revealed that these proteins were primarily enriched in complement–coagulation cascades and complement regulation pathways. Platelet activation, signaling, and aggregation pathways were also enriched (Figure S5C,D). The results of the Wiki pathway enrichment analysis are presented in Figure S5E, showing infection- and neurology-related pathways. GO annotation indicated the complement-related functions and blood coagulation function of these enriched proteins (Figure S5F).

For the 34 DEPs in KD patients with giant CAAs, the top DEPs were CLC, IG, etc. (Figure S6A). Upregulated proteins were related to the metabolism of proteins, and downregulated proteins were linked to the innate immune system and MAPK signaling. KEGG and Wiki pathway enrichment analysis revealed that these proteins were mainly enriched in cell function and gene modification pathways. The Rap1 signaling pathway was found to be enriched, suggesting that the DEPs in this pathway might contribute to the formation of giant CAAs (Figure S6B,C). The results of the Reactome Pathway enrichment analysis are presented in Figure S6D, showing complement–coagulation cascades and COVID-19 thrombosis pathways. GO annotation indicated the complement-related functions and cytoskeleton transport functions that these proteins enriched (Figure S6E).

These results revealed the proteomic profile of DEPs in KD patients with different severities of CAAs, suggesting the pathological mechanisms of small and medium CAAs related to complement and platelet activation, while the pathological mechanisms of giant CAAs are related to cell function pathways and the Rap1 signaling pathway.

## 3. Discussion

The present study investigated a comprehensive exosomal proteomic profile in KD patients, especially in patients with CAAs. Complement-related pathways and G-protein-related pathways were mainly enriched in KD patients compared with HC and FC groups. A WB analysis of S100A9 revealed significant differences in KD patients compared with the FC group. A further WB analysis of SERPINA1 and GNAI2 revealed significant differences in the KD groups with and without CAAs.

S100A9 is related to immune functions and inflammatory responses, indicating that it may be related to the pathology of KD [31,32,33]. Previous studies have demonstrated that S100A9 expression was elevated in untreated KD patients [34,35,36]. In our study, S100A9 was one of the top 10 DEPs in KD and FC patients, and its expression was also found to be significantly lower in KD patients compared with the HC and FC groups. These findings differ from the previously reported trend in KD serum S100A9 levels. This may be due to the interaction of S100A9 and fibrinogen, or the secretion of exosomal S100A9 into peripheral blood [33,37].

When we further focused on the DEPs in KD patents with and without CAAs, we found that the complement-related pathways were abundantly enriched, including the complement and coagulation cascade pathways, and we observed regulation of the complement cascade pathway, complement activation pathway, classical pathway, etc. These complement pathway results were consistent with the consensus that KD is an autoimmune disease. The pathways included various complement proteins that could trigger a cascade of downstream inflammatory factors, thereby contributing to disease progression. In the groups we analyzed, the top 10 proteins included complement proteins, such as the SERPINA family, C3, CFHR3, and CFHR4. These findings help in the selection of pathways and DEPs for further investigation.

Previous studies suggested the involvement of complement proteins in KD and infectious immune disorders [38,39]. Complement factors and their binding G-proteins can interact with the inflammatory factor NLR family pyrin domain containing 3 (NLRP3), which is a key proinflammatory component in the body [40,41]. Our study emphasized the complement-related pathway-enriched protein SERPINA1, which was also one of the top 10 DEPs in KD patients. SERPINA1 is a short peptide from α-1-antitrypsin (AAT). It is an acute-phase glycoprotein and plays a critical role both in activating and sustaining inflammatory cascades, as well as in anti-inflammatory action under different conditions [42,43]. A former study showed that SERPINA1 regulated MMP protein expression and was downregulated in the resolution phase of acute inflammation [43]. The significance of AAT in KD patients compared with febrile patients was highlighted, as it was a significantly altered protein within the complement pathways, suggesting its involvement in the pathogenesis of KD [44]. Additionally, studies have shown that AAT levels were lower in KD patients than in febrile patients, indicating that AAT and its peptide, SERPINA1, might play a role in the development of KD [45]. Furthermore, when comparing KD patients with and without CAAs, AAT expression was found to be downregulated in those with CAAs [46]. Collectively, these findings provide evidence of the relationship between the SERPINA1 gene and the development of KD.

We also focus on the role of SERPIN family members in KD and aneurysm development. Members of the Serpin family may play a role in the development of artery lesions and aneurysms. Serum protein SERPINE1 has been identified as a potential marker associated with CALs in KD [15]. SERPINC1 and fibronectin 1 have been recognized as significant genes involved in the pathogenesis of CALs in KD [47]. In addition, in tissue samples from abdominal aortic aneurysms (AAAs), an altered expression of SERPINA4 has been observed [48]. Furthermore, deficiencies in anti-SERPINA protein might be a risk factor for AAA [49].

Thus far, the relationship between SERPINA1 and CAAs has not been reported. We found that SERPINA1 was enriched in complement and coagulation cascade pathways and innate immune system pathways. Our study showed that the expression level of SERPINA1 was lower in KD patients with vs. without CAAs via LC-MS/MS and WB analysis, which may be due to the different roles it plays in KD with and without CAAs. Considering that the plasma exosome cargos may be released, the mechanism of SERPINA1 in KD inflammation needs to be further explored.

In addition, among the top 10 DEPs in our study, C3, CFHR3, and CFHR4 are crucial components of the body’s innate immune defenses [50,51]. Studies have demonstrated that the plasma level of C3 in KD patients was significantly elevated compared with FC and HC children [52,53]. In our study, C3 was mainly enriched in complement-related pathways and innate immune system pathways in KD patients, aligning with the pattern typically seen in inflammatory responses. CFHR3 and CFHR4 can lead to the secretion of proinflammatory cytokines such as interleukin-1β (IL-1β), IL-18, IL-6, and tumor necrosis factor-a (TNF-a). Given that IL-1β serves as an inflammatory marker of KD, the CFHR family may enhance the pathological effects associated with KD [54]. Similarly to C3, CFHR3 and CFHR4 were mainly enriched in complement-related pathways and innate immune system pathways, indicating that it may play an inflammation regulatory role in KD.

Beyond the complement pathways, our study identified a significant enrichment of G-protein-related pathways in KD patients, including the G-protein-coupled receptor signaling pathway, adenylate cyclase-activating G-protein coupled receptor signaling pathway, G-protein beta/gamma-subunit complex binding pathways, etc. The G-protein family is a crucial family in the physiological and pathological processes of the body, with their pathways involved in various responses. GNAI proteins are key components of the G-protein pathway and are abundantly expressed in granulocytes, lymphocytes, and endothelial cells [55]. The GNAI proteins can modulate the expression of TNF-α and IL-10 [56]. These functions of GNAI proteins suggest they may play a significant role in inflammatory responses in KD.

In our study, we found that GNAI2 was expressed as a significantly changed DEP associated with increased disease severity, which was related to the G-protein pathways in KD patients. The guanine nucleotide-binding G-protein G(i) subunit alpha-2, GNAI2, is a component of heterotrimeric G-proteins. It is involved in both GPCR and non-GPCR signaling pathways [57,58,59]. GNAI2 is crucial protein in regulating neutrophil transport and is essential for chemokine-induced neutrophil arrest [60]. It can pair with the chemokine receptor to regulate the body’s responses [61]. These functions imply that GNAI2 may play a role in triggering the acute phase of KD.

Furthermore, GNAI2-associated TNF-α and chemokine may play a role in CAAs. During aneurysm formation, TNF-α is involved in the inflammatory response of vascular smooth muscle cells (VSMCs), mediating aneurysmal lesions [62,63]. Compared with patients without lesions, those with coronary artery ectasia exhibited significantly elevated levels of TNF-α expression [64]. In GNAI2 knockout mice, an increased expression of TNF-α has been observed, which may explain the altered role of GNAI in CAAs among children with KD. C-X-C motif chemokine ligands (CXCLs) demonstrate high levels of intercellular communication signaling in aortic aneurysms [65], and blocking the binding of CXCL2 to receptor CXCR2 can significantly reduce the extent of aortic dilation [66]. These findings suggest a potential mechanism by which GNAI2 alterations contribute to CAA. It was notable that GNAI2 was found to be the most enriched among all the top 20 KEGG pathways of the significantly changed DEPs in KD patients. Additionally, pathways related to G-proteins and GTPase were also abundantly enriched in DEPs specific to KD patients, highlighting the importance of investigating the role of GNAI2. The LC-MS/MS result and WB analysis showed that the expression level of GNAI2 was lower in KD patients with vs. without CAAs. This may be on account of the neutrophil aggregation and proinflammatory effects in the acute phase of KD.

In addition to the highlighted pathways described above, the platelet activation pathway was also enriched in DEPs in KD patients compared with HC children. An elevation in platelet count and a hypercoagulable state exist in KD patients with CAAs. Our laboratory data showed that the platelet level in KD patients with CAAs was significantly increased, and there was an increasing trend in the aggravation of the severity of CAAs, indicating that the DEPs linked to the activation of platelets might participate in KD with CAAs and even in CAAs of different severities, which needs to be further investigated.

Last but not least, previous studies utilized 2-DE for the proteomic analysis of exosomes in KD [28,29,30]. By contrast, our study employed LC-MS/MS with TMT6 labeling to identify DEPs, offering higher sensitivity and resolution. This approach is increasingly favored for protein identification.

There are limitations to the present study. A large sample of patients with CAAs is necessary to validate our findings in future studies. A clinical study should be designed to evaluate the efficacy of these potential biomarkers. Further research is needed to explore the association of DEPs we selected, and the pathogenesis of KD and CAAs in cells and animal models.

## 4. Materials and Methods

### 4.1. Recruited Subjects

A total of 60 subjects were included in this study, with 40 subjects studied via liquid chromatography–tandem mass spectrometry (LC-MS/MS) with tandem mass tag (TMT)-labeled prometric analysis, and the other 20 subjects undergoing Western blotting (WB) validation. In the discovery group, 24 KD patients were recruited from the Cardiovascular Medicine Department of the Children’s Hospital Capital Institute of Pediatrics during the period from January 2019 to January 2024, with 8 age- and sex-matched healthy control (HC) children recruited from the Medical Care Department and 8 age- and sex-matched febrile control (FC) patients recruited from the Respiratory Medicine Department of the same hospital during the same period. In the validation group, an additional 12 KD patients were recruited, with an additional 4 HC children and 4 FC patients from the same hospital during the same period. The controls were age- and sex-matched as well, as shown in Table 1.

The diagnosis of KD was based on the guidelines published by the American Heart Association (AHA) in 2017 [1]. CALs were identified based on the maximum internal diameter of the coronary arteries measured by echocardiographic assessment. The severity of CALs was classified by the Z-scores calculated by the Kabayashi model: (1) no involvement: always <2; (2) dilation only: 2 to <2.5, or if initially <2, a decrease in Z score was observed during follow-up ≥1; (3) small aneurysm: ≥2.5 to <5; (4) medium aneurysm: ≥5 to <10, and absolute dimension <8 mm; and (5) giant aneurysm: ≥10, or absolute dimension ≥8 mm. The maximum Z score (Zmax) was defined as the largest Z score of the LMCA, LAD, LCX, or RCA on echocardiography.

Based on the Z-scores, 24 KD patients in the discovery group and 12 KD patients in the validation group were further equally divided into three subgroups. The KD-1 group consisted of patients without CAAs, the KD-2 group included those with small and medium CAAs, and the KD-3 group comprised patients with giant CAAs.

The children in the HC group showed no signs of infection at the time of recruitment. The major clinical manifestation in children in the FC group was a temperature > 38 °C at the time of recruitment. Laboratory tests in the FC group showed a change in white blood cell and lymphocyte numbers. Clear pathogenic evidence was observed in FC patients, such as bacteria, viruses, or mycoplasmas. No FC patients met the diagnostic criteria for KD or other rheumatic immune diseases.

### 4.2. Collection and Isolation of Plasma Exosome Samples

Blood samples were collected from KD patients after the diagnoses were confirmed and before the IVIG treatment. Similarly, blood samples were obtained from febrile patients prior to antibiotic treatment.

Approximately 1.5 mL of blood was harvested via venipuncture into tubes containing EDTA as an anticoagulant. These blood samples were centrifuged at 4 °C, 2000× *g*, for 5 min to separate approximately 0.6 to 1 mL of upper plasma. The plasma was then aliquoted and stored at −80 °C until further processing. For further operation, 2 mL of filtered 1× PBS was added to 400 µL of clarified plasma, achieving a 6-fold dilution. The mixture was vortexed and centrifuged at 300× *g* for 10 min at room temperature. The supernatant was collected and subjected to another centrifugation at 2000× *g* for 20 min at room temperature. The resulting supernatant was again collected and centrifuged at 10,000× *g* for 30 min at room temperature. The final supernatant underwent ultracentrifugation at 4 °C, at 100,000× *g*, for 70 min. The supernatant was carefully aspirated with a pipette, and the ultracentrifugation process was repeated with filtered 1× PBS in the centrifuge tube. Finally, filtered 1× PBS was added to the exosome suspension, which was mixed gently with a pipette. The exosome suspension was then ready for downstream analysis.

### 4.3. Preparation of Plasma Tryptic Digests and TMT6 Labeling

Exosomes extracted from the eight individuals in each subgroup were pooled with equal quality. Prior to analysis, the samples underwent enzymatic digestion with trypsin. Approximately 100 µg of exosomal proteins were denatured using 8 M urea and reduced with 10 mM dithiothreitol at 37 °C for one hour. Following this, cysteine residues were alkylated with 40 mM iodoacetamide in the dark for one hour at 37 °C. The samples were then desalted using PD-10 columns. Trypsin was added at an enzyme-to-protein ratio of 1:50, and the mixture was incubated at 37 °C overnight. All procedures were mixed at 800 rpm. The digested samples were subsequently purified using C-18 solid-phase extraction tubes. To enhance the detection and quantification of exosomal proteins, two replicates labeled with TMT6 were produced.

### 4.4. Mass Spectrometry Data Analysis

Samples were analyzed using a QExactive HF mass spectrometer (Thermo Scientific, Waltham, MA, USA) coupled with an UltiMate 3000 RSLC nano System (Dionex, Sunnyvale, CA, USA). Mass spectrometry imaging (MSI) data were acquired in the Orbitrap at a resolution of 120,000, covering a mass-to-charge (*m*/*z*) range of 350 to 1350, with a maximum injection time of 100 milliseconds. Ions with charge states from 2 to 6 were selected for sequencing, utilizing a dynamic exclusion window of 60 s to exclude isotopic peaks. MS2 sequencing was performed in the ion trap after quadrupole selection and collision-induced dissociation (CID) fragmentation, within an *m*/*z* range of 400 to 2000. The resulting raw data were processed using Peaks X Studio (Version 10.0, Bioinformatics Solutions Inc., Waterloo, ON, Canada), with a search conducted against a Homo sapiens database.

### 4.5. Bioinformatics Analysis

After removing duplicates, unnamed proteins, and those with missing kurtosis values, the remaining proteins were subjected to bioinformatics analysis. Proteins in both the KD and FC groups, as well as those associated with different severities of CAAs in KD patients, were selected for further analysis. The DEPs were defined based on a fold change greater than 1.3. Statistical difference was determined by an adjusted *p*-value less than 0.05. Pathway analyses, including KEGG (Kyoto Encyclopedia of Genes and Genomes) pathway analysis, Reactome pathway analysis, Wiki pathway analysis, and Gene ontology (GO) term annotations for DEPs in exosomes isolated from children plasma, were conducted by DAVID Bioinformatics: “https://david.ncifcrf.gov/summary.jsp (accessed on 1 March 2024)”. Trend analysis was performed by Short Time-series Expression Miner (STEM) software (Version 1.3.8, Carnegie Mellon University, Pittsburgh, PA, USA). The protein–protein interaction (PPI) network was constructed by STRING Version 12.0: “https://cn.string-db.org/ (accessed on 1 May 2024)”.

### 4.6. Western Blotting

The DEPs analyzed were validated using WB. The exosomal proteins were separated on a 4–20% SDS-PAGE gel and transferred onto nitrocellulose (NC) membranes. The membranes were blocked with 10% milk for 1 h and then incubated overnight at 4 °C with primary antibodies, including S100A9 (Proteintech, Wuhan, China, #26992-1-AP), GNAI2 (Proteintech, Wuhan, China,#11136-1-AP), and SERPINA1 (Proteintech, Wuhan, China,#66135-1-Ig). After 12 h, the membranes were washed with PBS containing Tween 20 and then incubated with anti-rabbit/mouse IgG secondary antibody at room temperature for 1 h. After being washed again, the protein bands were visualized using the Image Lab system (Gel Image System, Tanon, Shanghai, China), and their expression levels were quantified by relative α-Tubulin (CST, Danvers, MA, USA, #2148S) staining.

### 4.7. Statistical Analysis

Data analysis was conducted using SPSS software, version 23. All datasets were evaluated for normality and homogeneity of variance. For datasets that followed a normal distribution and demonstrated homogeneity of variance, data are expressed as the average ± SD, Student’s t-test was used for comparisons between two groups, and ANOVA was applied for comparisons among multiple groups. When the data did not meet the criteria for normality or exhibited unequal variances, data are expressed as the median with quartile range (M (Q1, Q3)), and non-parametric tests were employed to assess statistical differences. The chi-square test and Fisher’s exact test were conducted for categorical variables. A *p*-value of less than 0.05 was considered statistically significant. In figures, significance levels are denoted as follows: *p* < 0.05 by one asterisk (*).

## 5. Conclusions

In conclusion, SERPINA1 and GNAI2 were selected from the comprehensive proteomic profile in KD patients to explore their differences in KD patients with and without CAAs. SERPINA1 and GNAI2 were primarily enriched in the complement-related pathways, immune system pathways, and G-protein-related pathways, which may be linked to the pathological mechanisms of KD and CAAs. Consequently, these proteins and pathways provide a novel perspective for exploring the mechanisms underlying KD with CAAs, and they also might serve as promising biomarkers for KD with CAAs.

## Figures and Tables

**Table 1:** Baseline characteristics of the patients with Kawasaki disease and the controls.

	Control Groups				KD Groups					
	Discovery Group		Validation Group		Discovery Group			Validation Group		
Subgroups	HC	FC	HC	FC	KD-1	KD-2	KD-3	KD-1	KD-2	KD-3
No. of participants	8	8	4	4	8	8	8	4	4	4
Age (year)	2.14 ± 1.28	1.92 ± 1.04	2.25 ± 1.86	1.73 ± 1.76	2.09 ± 1.37	2.45 ± 1.47	2.20 ± 1.43	2.1 ± 1.62	1.82 ± 1.65	2.42 ± 1.29
Males/Females, (n)	4/4	4/4	3/4	3/4	4/4	4/4	4/4	3/4	3/4	3/4
Z score	/	/	/	/	/	5.87 ± 2.87	13.23 ± 2.63	/	6.23 ± 2.85	11.96 ± 2.09
WBC, ×10^9^/L					13.01 ± 4.83	12.89 ± 6.78	10.83 ± 5.33	14.97 ± 2.66	13.21 ± 2.60	10.76 ± 4.60
NEUT, ×10^9^/L					8.15 ± 3.02	7.49 ± 4.73	6.31 ± 5.02	9.05 ± 3.15	7.29 ± 3.35	7.32 ± 3.89
PLT, ×10^9^/L					339.63 ± 102.75	487.00 ± 210.05	625.00 ± 267.02	513.00 ± 62.80	569.75 ± 152.99	371.75 ± 171.93
CRP, mg/L					59.60 ± 32.28	55.08 ± 51.67	15.50 (1, 38)	34.64 ± 23.56	52.65 ± 51.73	93.70 ± 79.29

Discovery group: patients of exosome proteomics LC-MS/MS-TMT6 detection; validation group: patients of WB analysis of selected DEPs. KD: Kawasaki disease; KD-1: KD patients without CAAs; KD-2: KD patients with small and medium CAAs; KD-3: KD patients with giant CAAs; HC: healthy control; FC: febrile control.

**Table 2:** Laboratory data of the KD patients without and with CAAs.

	KD Groups			
	Discovery Group		Validation Group	
Subgroups	KD-1	KD-2 + KD-3	KD-1	KD-2 + KD-3
WBC, ×10^9^/L	13.01 ± 4.83	11.86 ± 5.99	14.97 ± 2.66	11.98 ± 3.70
NEUT, ×10^9^/L	8.15 ± 3.02	6.90 ± 4.75	9.05 ± 3.15	7.30 ± 3.36
PLT, ×10^9^/L	339.63 ± 102.75	556.00 ± 210.05	513.00 ± 62.80	470.75 ± 184.12
CRP, mg/L	59.60 ± 32.28	24.50 (2.83, 65.89)	34.64 ± 23.56	73.18 ± 65.75

Discovery group: patients of exosome proteomics LC-MS/MS-TMT6 detection; validation group: patients of WB analysis of selected DEPs. KD: Kawasaki disease; KD-1: KD patients without CAAs; KD-2 + KD-3: KD patients with CAAs.

## Data Availability

The authors declare that the data supporting the findings of this study are available within this paper. Should any raw data files be needed, they are available from the corresponding author upon reasonable request.

## Supplementary Materials

The supporting information can be downloaded at: https://www.mdpi.com/article/10.3390/ijms26062668/s1.

## Abbreviations

KD, Kawasaki disease; CAAs, coronary artery aneurysms; CADs, coronary artery dilatations; CALs, coronary artery lesions; DEP, differentially expressed protein; LC-MS/MS, liquid chromatography–tandem mass spectrometry; WB, Western blotting; IVIG, intravenous immunoglobulin; 2-DE, two-dimensional electrophoresis; HC, healthy control; FC, febrile control; WBC, white blood cell; NEUT, neutrophil; PLT, platelet; CRP, C-reactive protein. SERPINA1, α-1-antitrypsin short peptide; GNAI2, guanine nucleotide-binding protein G(i) subunit alpha-2.
