# Supplementary material for: Plasma Exosomal-Derived SERPINA1 and GNAI2 Downregulation as Potential Diagnostic Biomarkers of Kawasaki Disease with Coronary Artery Aneurysms
Source: Int J Mol Sci. 2025 Mar 16;26(6):2668. doi: 10.3390/ijms26062668 (PMC11942354; doi:10.3390/ijms26062668)
Supplement: Supplementary file 1 [file ijms-26-02668-s001.zip › ijms-3482773-supplementary.pdf]

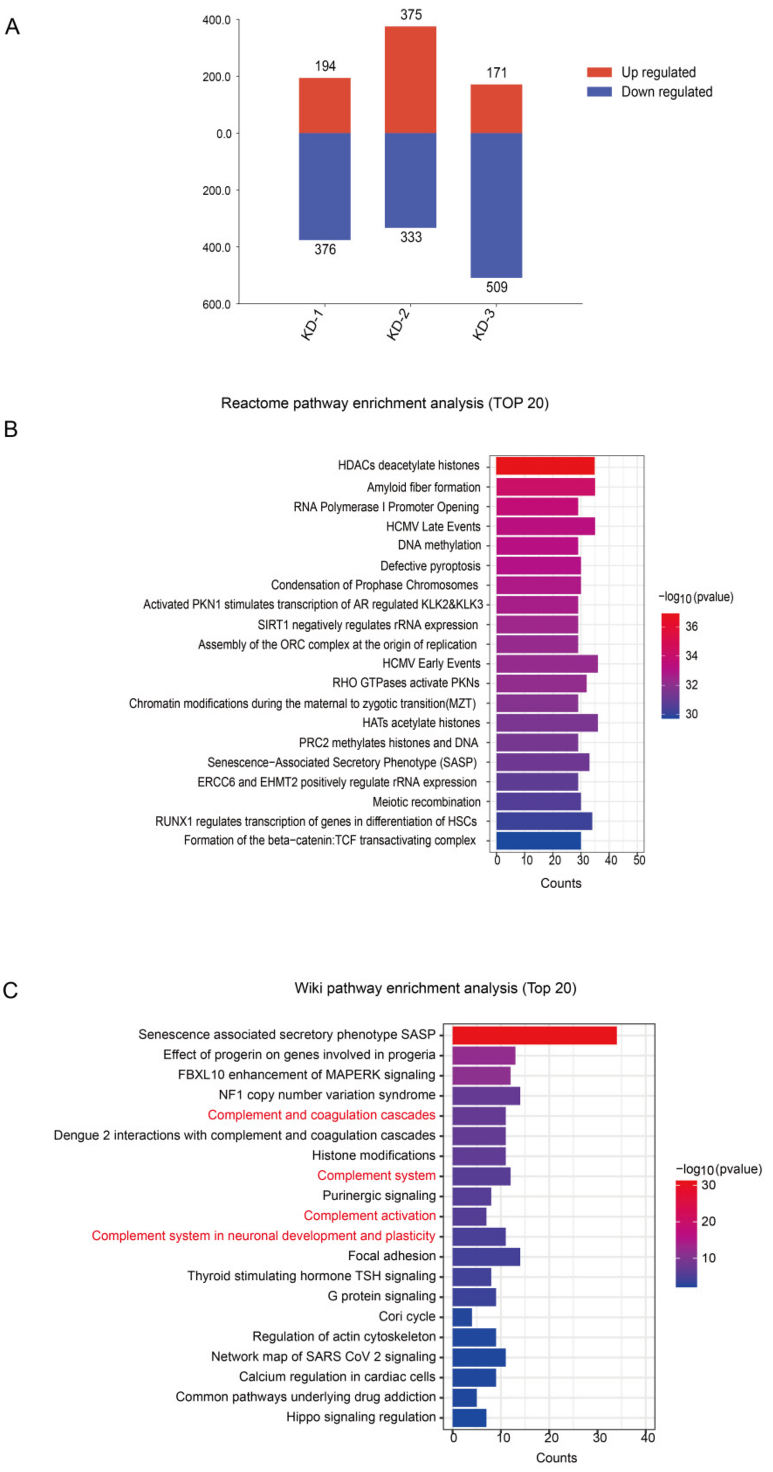

Figure S1 Supplementary protein expression profiles of plasma exosomes in KD patients compared with HC group

(A)Bar graph showed the number of upregulated and downregulated DEPs in KD patients compared with HC group.

(B)Reactome pathway enrichment analysis of 332 exosomal DEPs expressed both in KD patients, showing the top 20 enriched pathways.

(C) Wiki pathway enrichment analysis of 332 exosomal DEPs expressed in KD patients, showing the top 20 enriched pathways.

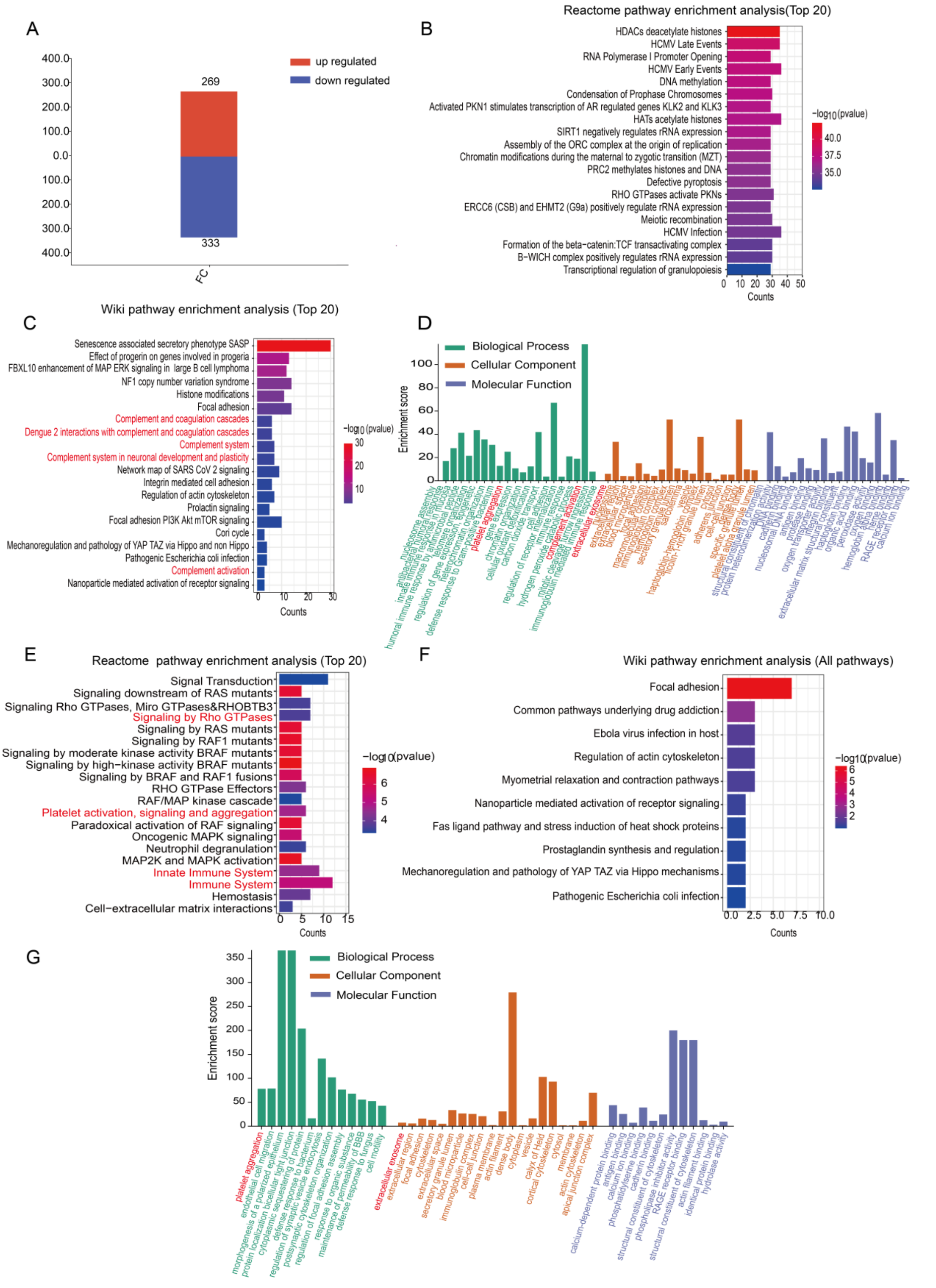

Figure S2 Supplementary protein expression profiles of plasma exosomes expressed in KD patients compared with FC group

(A)Bar graph showed the number of upregulated and downregulated DEPs in FC group compared with

HC group

(B) Reactome pathway enrichment analysis of 228 exosomal DEPs expressed in KD patients compared with FC group, showing the top 20 enriched pathways.

(C)Wiki pathway enrichment analysis of 228 exosomal DEPs expressed in KD patients compared with FC group, showing the top 20 enriched pathways.

(D) GO annotations for 228 exosomal DEPs in KD patients compared with FC group.

(E) Reactome pathway enrichment analysis of 24 down-regulated exosomal DEPs expressed in KD patients compared with FC group, showing the top 20 enriched pathways.

(F)Wiki pathway enrichment analysis of 24 down-regulated exosomal DEPs expressed in KD patients compared with FC group, showing the all enriched pathways.

(G) GO annotations for 24 down-regulated exosomal DEPs in Profiles 0.

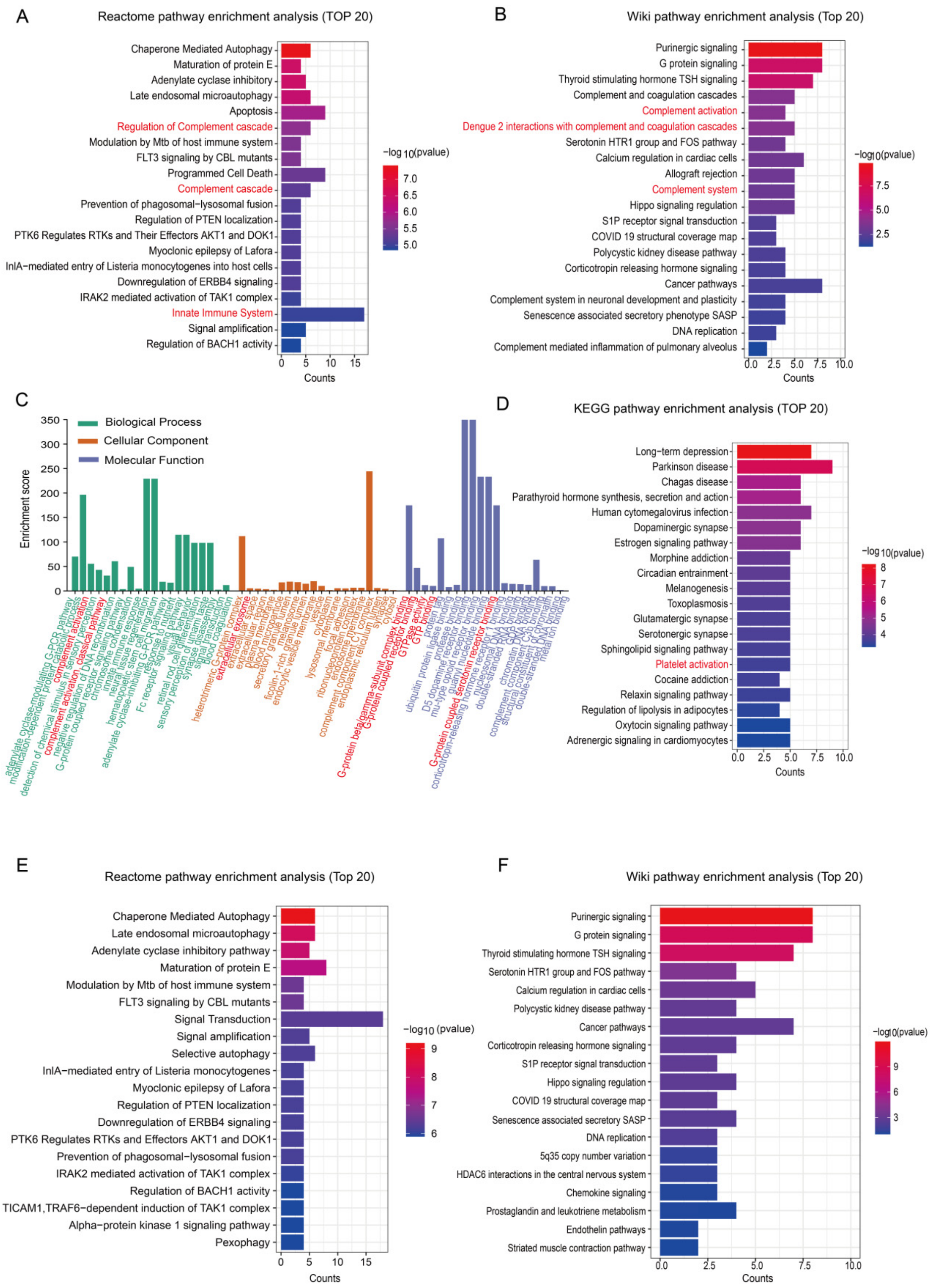

Figure S3 Supplementary protein expression profiles of plasma exosomes expressed in KD without CAAs and KD with CAAs groups

(A) Reactome pathway enrichment analysis of 104 expressed exosomal DEPs in KD patients, showing the top 20 enriched pathways.

(B) Wiki pathway enrichment analysis of 104 uniquely expressed exosomal DEPs in KD patients, showing the top 20 enriched pathways.

(C)GO annotations for 104 exosomal DEPs uniquely expressed in KD patients.

(D) KEGG pathway enrichment analysis of 54 exosomal DEPs in Profile 0, showing the top 20 enriched pathways.

(E) Reactme pathway enrichment analysis of 54 exosomal DEPs in Profile 0, showing the top 20 enriched pathways.

(F)Wiki pathway enrichment analysis of 54 exosomal DEPs in Profile 0, showing the top 20 enriched pathways

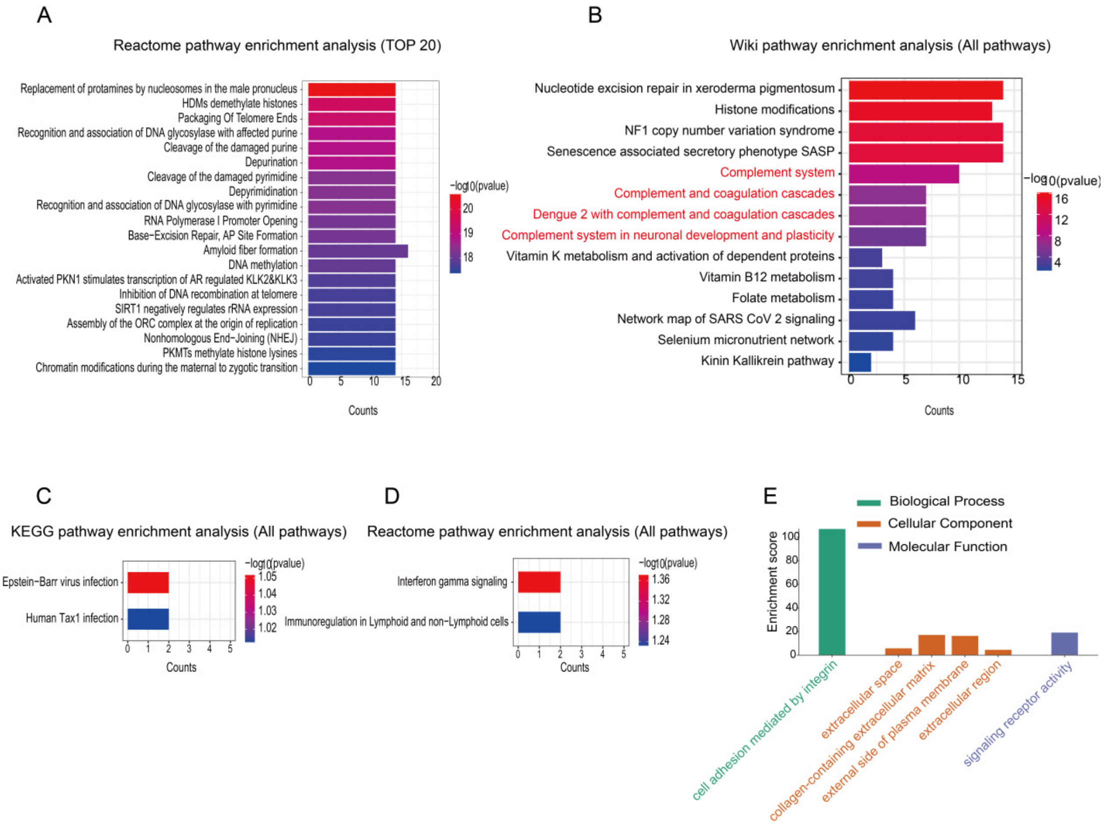

Figure S4　Supplementary protein expression profiles of plasma exosomes in KD without CAAs and KD with CAAs groups separately

(A) Reactome pathway enrichment analysis of 91 exosomal DEPs expressed in KD-2+KD-3 groups, showing the top 20 enriched pathways

(B) Wiki pathway enrichment analysis of 91 exosomal DEPs expressed in KD-2+KD-3 groups, showing all enriched pathways.

(C) KEGG pathway enrichment analysis of 16 exosomal DEPs expressed in KD-1 group, showing all enriched pathways.

(D) Reactome pathway enrichment analysis of 16 exosomal DEPs expressed in KD-1 group, showing all enriched pathways.

(E) GO annotations for 16 exosomal DEPs expressed in KD-1 group.

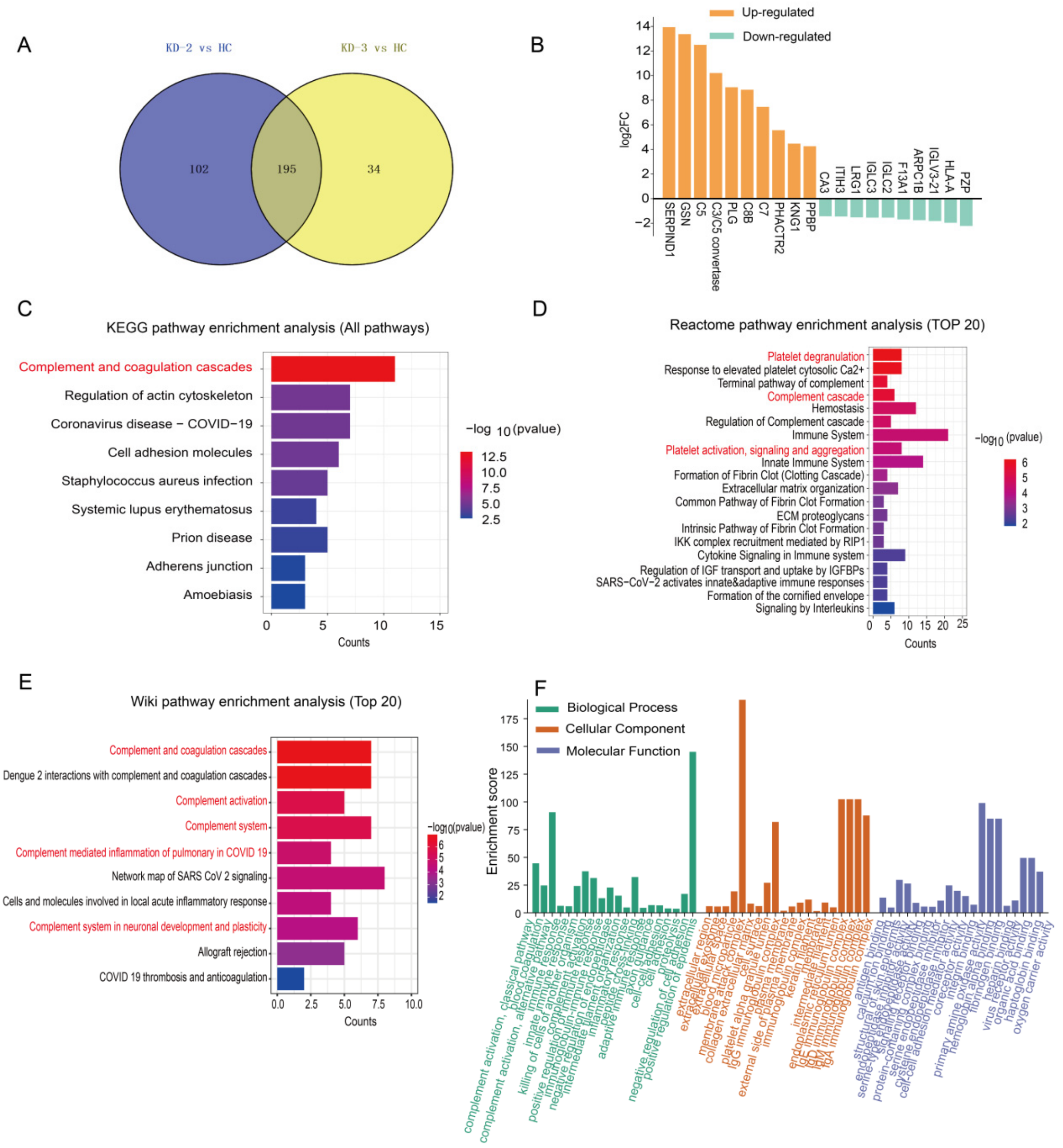

Figure S5 Supplementary protein expression profiles of plasma exosomes in KD patients with small and medium CAAs

(A) Venn diagram showed the number of DEPs in KD patients with small and meduim CAAs and with giant CAAs.

(B)Top 10 upregulated/downregulated proteins in KD patients with small and medium CAAs.

(C) KEGG pathway enrichment analysis of 102 exosomal DEPs expressed in KD patients with small and medium CAAs, showing all enriched pathways.

(D) Reactome pathway enrichment analysis of 102 exosomal DEPs expressed in KD patients with small and medium CAAs, showing the top 20 enriched pathways.

(E) Wiki pathway enrichment analysis of 102 expressed in KD patients with small and medium CAAs, showing the top 20 enriched pathways.

(F) GO annotations for 102 exosomal DEPs expressed in KD patients with small and medium CAAs.

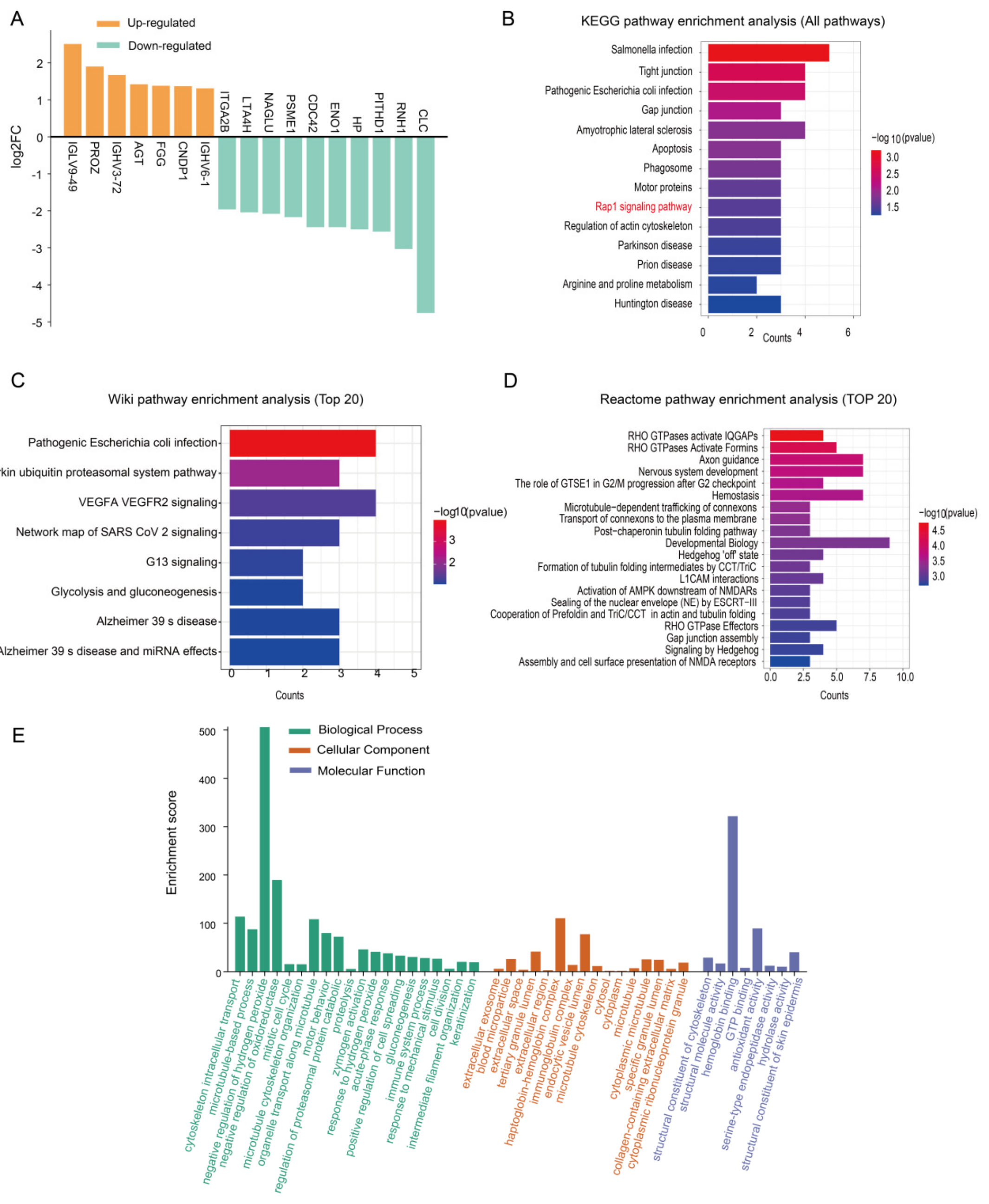

Figure S6 Supplementary protein expression profiles of plasma exosomes in KD patients with giant CAAs

(A)Top upregulated/downregulated proteins in KD patients with giant CAAs, showing all upregulated DEPs and top 10 downregulated DEPs.

(B) KEGG pathway enrichment analysis of 34 exosomal DEPs expressed in KD patients with giant CAAs, showing all enriched pathways.

(C) Wiki pathway enrichment analysis of 34 exosomal DEPs expressed in KD patients with giant CAAs, showing the top 20 enriched pathways.

(D)Reactome pathway enrichment analysis of 34 exosomal DEPs expressed in KD patients with giant CAAs, showing the top 20 enriched pathways.

(E) GO annotations for 34 exosomal DEPs expressed in KD patients with giant CAAs.
